# Weight gain on dolutegravir: Association is not the same as causation

**DOI:** 10.4102/sajhivmed.v24i1.1500

**Published:** 2023-05-15

**Authors:** Gary Maartens, Phumla Sinxadi, W.D. Francois Venter

**Affiliations:** 1 Division of Clinical Pharmacology, Department of Medicine, Faculty of Health Sciences, University of Cape Town, Cape Town, South Africa; 2 Ezintsha, Faculty of Health Sciences, University of the Witwatersrand, Johannesburg, South Africa

Dolutegravir and other integrase strand transfer inhibitors are associated with more weight gain in people starting antiretroviral therapy (ART) than other antiretroviral drug classes.^1,2^ Antiretroviral therapy-naïve people gain weight after starting ART, irrespective of whether the regimen includes dolutegravir – this is a return-to-health phenomenon. Weight gain is most marked if ART is started with CD4 counts below 200 cells/μL.^1^ Women gain more weight after starting ART than men.^1^ The South African ADVANCE study reported more weight gain in participants randomised to dolutegravir than efavirenz, which was more marked among women.^3^ This observation has caused concern because women in sub-Saharan Africa have a higher prevalence of both HIV and obesity than men.^4^

There are two hypotheses for the greater weight gain observed with dolutegravir than efavirenz: dolutegravir may be causing weight gain or efavirenz may be impairing weight gain. There are no credible mechanisms by which dolutegravir could cause weight gain. Integrase strand transfer inhibitors inhibit the human melanocortin 4 receptors in vitro, which plays a role in the control of appetite in the brain, but inhibition only occurs at concentrations far exceeding those that are achievable clinically. By contrast, there are biologically plausible reasons why efavirenz could impair weight gain: by impairing appetite from chronic neuropsychiatric effects or by its toxic effects on adipocytes.^5,6^

Recent studies provide very strong evidence for the hypothesis that efavirenz impairs weight gain in genetically susceptible individuals. Efavirenz concentrations are highly variable, which is mainly due to loss-of-function mutations in genes encoding for the cytochrome P450 enzymes responsible for metabolising efavirenz, CYP2B6 (major pathway) and CYP2A6 (minor pathway). Mutations in these genes are used to categorise people into extensive, intermediate, and slow efavirenz metabolisers – intermediate metabolisers have modestly increased efavirenz concentrations while slow metabolisers (about 20% of South Africans) have markedly increased efavirenz concentrations.^7^ A genetic sub-study in ADVANCE participants randomised to the efavirenz arm found that efavirenz metaboliser genotype predicted weight gain to 48 weeks: extensive metabolisers gained the most weight, followed by intermediate metabolisers, then slow metabolisers, who actually experienced a slight decline from baseline in median weight.^8^ A key point was that weight gain was similar between extensive metabolisers in the efavirenz arm and in the dolutegravir arm with the same nucleoside reverse transcriptase inhibitor backbone (tenofovir disoproxil fumarate and emtricitabine). A cohort study reported that efavirenz slow metabolisers gained the most weight after switching from efavirenz to integrase inhibitors when virologically suppressed, providing further evidence for impaired weight gain on efavirenz-based ART in slow metabolisers.^9^

The belief that dolutegravir causes weight gain is widespread among people with HIV and clinicians, which can result in inappropriate antiretroviral switching, reduced adherence, and failure to manage people who are overweight and obese with appropriate interventions.

People with marked weight gain on dolutegravir-based ART should be screened for the metabolic syndrome and treated accordingly. The weight gain should be addressed by appropriate lifestyle and other interventions. Newer interventions are more effective at maintaining weight loss for people with established obesity than lifestyle measures but access to these interventions is limited in our region, especially in the public sector. Switching antiretrovirals in people who experience weight gain on dolutegravir is recommended against in the 2023 guidelines from the Southern African HIV Clinicians Society and the National Department of Health. Switching from dolutegravir to efavirenz could result in weight loss in efavirenz slow metabolisers, but they are at increased risk of severe toxicity, including drug-induced liver-injury and chronic neuropsychiatric conditions.^5^
